# University Students' Self-Medication Practices and Pharmacists' Role: A Cross-Sectional Survey in Hail, Saudi Arabia

**DOI:** 10.3389/fpubh.2021.779107

**Published:** 2021-12-17

**Authors:** Farhan Alshammari, Ahmed Alobaida, Abdulhadi Alshammari, Atheer Alharbi, Adel Alrashidi, Asma Almansour, Amal Alremal, Kashif Ullah Khan

**Affiliations:** ^1^Department of Pharmaceutics, College of Pharmacy, University of Hail, Hail, Saudi Arabia; ^2^Department of Clinical Pharmacy, College of Pharmacy, University of Hail, Hail, Saudi Arabia

**Keywords:** students, pharmacists, Hail University, Saudi Arabia, self-medication

## Abstract

**Background:** Self-medication is an important issue for health authorities around the world. It is also a common practice among university students.

**Objective:** This study aimed to assess the prevalence of and reasons for self-medication among university students.

**Methods:** A descriptive, cross-sectional community based survey design was adopted for the current study. All the students enrolled in Hail University, Saudi Arabia were selected to include in the study. Data was collected from February to April 2020 using a validated questionnaire and were analyzed using IBM SPSS Statistics, Version 22.0. Using convenient sampling technique, the total sample size calculated was 370 participants. A descriptive analysis was performed. Chi-square test and binary logistic regression was used for analyzing the data where statistical significance was set at *p* ≤ 0.05.

**Results:** A total of 373 participants completed the questionnaire. The response rate was 84%. The overall prevalence of self-medication among the students was 98.2%. Of the 373 participants, 40.8% were men, and 59.2% were women. Furthermore, 56% were in fields other than health sciences, 23.1% were in preparatory foundation courses, and 20.9% were from the health and medical sciences. The most frequent medical condition that led to self-medication was headaches (92.85%), followed by coughs (37.5%), colic (31.9%) and influenza (30.3%). On univariate analysis, it is revealed that for both abdominal colic [OR 0.54 (0.34–0.86), *p* = 0.01] and constipation [OR 0.57 (0.32–1.02), *p* 0.05], female gender was observed significantly with low prevalence than male. However, for influenza, the self-medication prevalence [OR 1.86 (1.19–2.91), *p* = 0.006] observed was significantly higher in female participants than male. A significant association (*p* = 0.011) between the self-medication factors and gender was shown in the current study results.

**Conclusion:** An alarming prevalence of self-medication among the students was recorded. Health care providers can increase awareness of the issue by educating individuals about the harmful effects of irresponsible self-medication.

## Introduction

Medication consumption trends are an important health predictor. The awareness of trends can lead to the identification and determination of disease prevalence, as well as the ability to gain insights into the use of clinical services (1). Self-medication is a major concern because it can hinder diagnosis and lead to the proliferation of resistant microorganisms and iatrogenic illnesses (2). Previous studies in various countries have reported an increase in self-medication (3), which has been described as a patient's decision to take medication on their own or on the advice of a pharmacist or layperson rather than consult a medical practitioner (1). Generally, over-the-counter drugs are involved, but prescription-only medicines have also been used (4, 5). Age, gender, financial position, self-care attitude, education level, medical literacy, happiness and non-seriousness of illness are influential factors in self-medication behaviors (6, 7).

Self-medication has been found to be very common among university students. Previous studies have reported a high self-medication prevalence (55–98%) among university students in Egypt and Palestine (8, 9). A high self-medication prevalence (86.6%) was found among male university students at Al-Qassim University (10). A study at Taibah University revealed that a considerably high proportion (64.8%) of students used non-prescription medications (11). Most medications can cause severe side effects that could have serious health implications and even be life-threatening. Therefore, a physician's evaluation is critical for effective care (12). Community pharmacists play a significant role in facilitating self-medication. Patients who can be prescribed medicines by pharmacists and those who should be advised to contact physicians should be differentiated by the pharmacist (12). According to the World Health Organization, pharmacists are central players in the supply and distribution of drugs to the public (13). In their clinical capacity, pharmacists are qualified to provide guidance on the medications they provide. Individuals are increasingly treating a significant number of illnesses without the aid of doctors or pharmacists. Pharmacists can play an important role in helping them to make well-informed self-care decisions (12).

Few studies have addressed the prevalence of self-medication among Saudi university students. The influential factors in self-medication behaviors are still being debated; thus, more research is needed. The study aimed to determine the prevalence of self-medication among Saudi university students. Self-medication for the most common disease states in north-eastern Saudi Arabia and pharmacists' involvement were assessed.

## Methodology

### Study Setting and Population

This study was based on a descriptive, cross-sectional community based survey. Current study was carried out in all the enrolled students of Hail University, Saudi Arabia, from February to April 2020. Hail, located in the north of the Kingdom of Saudi Arabia has an estimated population of 356,770 Saudi nationals and expatriates and a capital of Hail Region among 1 of the 13 regions of the Kingdom of Saudi Arabia. The University of Hail is a public institute located in main city of Hail where the student population is rated medium.

### Data Collection

The validated structured questionnaire contained total 31 close-ended questions with “yes and no response about self-medication practices and pharmacists” roles was adopted for the current study. The questionnaire had three sections. Part I elicited participant sociodemographic characteristics e.g., age, gender and nationality, university program and university course etc. Part II had two sub-parts, where subpart A contained 13 questions about the influential factors, including medical conditions, in self-medication, while subpart B had 04 questions about the factors that contribute to self-medication. Whereas, the third and final section had 6 questions used for evaluating the contribution of pharmacist in self-medication. The study questionnaire was adapted from various similar studies conducted previously and pre-tested on a sample of 10 participants, any ambiguities in the questions or responses were removed before its implementation (12, 14). In addition, the questionnaire was distributed to the male and female sections. Before the administration of the questionnaire, the students provided informed consent and received information about the study. The purpose of study was explained to all study participants and a consent form had been signed before the questionnaire would be given to students. Appropriate time were given to respondents to complete the questionnaire and return back. Students were with written informed consent and willing to participate in the study was included in study. To ensure diversity, all registered students, regardless of their social and demographic backgrounds, were eligible for inclusion in the study.

### Sampling Technique

Convenient sampling technique was used to collect data from study population. Sample size of current study was calculated based on Raosoft sample size calculator, Where margin of error is 5%, confidence level is 95%, population size of university students is 10,000 available at the university website and response distribution is 50%. The total sample calculated was 370 (15).

### Data Analysis

The data were analyzed using IBM SPSS Statistics, Version 22.0. A descriptive analysis was performed. The data are reported as percentages. The chi-square test was used, and statistical significance was set at *p* ≤ 0.05.

### Ethical Approval

The University of Hail research and ethics committee reviewed the research study proposal for any ethical issues. The study was approved by the committee (Nr. 41/213/43602). The students were given an assurance of confidentiality before being asked to provide written informed consent.

## Results

### Participant Demographics

A total of 444 students were approached, and 373 completed the questionnaire. The response rate was 84%. Most were women (59.2%), a majority (55.2%) of whom were aged 21–25 years. In addition, 97.6% were Saudi nationals. A majority (75.6%) of the participants were undergraduates (Table 1).

**Table 1:** Participant demographics (*N* = 373).

**Characteristics**	**Number (%)**
**Gender**	
Male	152 (40.8)
Female	221 (59.2)
**Age group**	
<20 years	150 (40.2)
21–25	206 (55.2)
26–30	11 (2.9)
>30	6 (1.6)
**Nationality**	
Saudi	364 (97.6)
Non-Saudi	9 (2.4)
**University program**	
Foundation	84 (22.5)
Undergraduate	282 (75.6)
Post-graduate	7 (1.9)
**University course**	
Health and medical science	78 (20.9)
Education	38 (10.2)
Preparatory	86 (23.1)
Computer science	21 (5.6)
Engineering	14 (3.8)
Art and literature	32 (8.6)
Business administration	73 (19.6)
Shari'a and law	7 (1.9)
Science	21 (5.6)
Community	3 (0.8)
**Residence**	
Off campus	371 (99.5)
On campus	2 (0.5)
Total response rate	84%

*Study sample response prevalence was recorded 84%*.

### Self-Medication Prevalence Among University Students

The most common symptoms and diseases reported by participants who practiced self-medication were headaches (92.8%, *n* = 346), coughs (37.5%, *n* =140), colic (31.9%; *n* =119) and influenza ([flu] 30.3%, *n* =113; Table 2). There was a statistically significant relationship between medical conditions, including flu and colic and gender (Table 2). The prevalence of self-medication for colic was higher among women (22%, *p* = 0.009); however, for flu (15.5%, *p* = 0.006), it was higher among men (Table 2). Upon binary logistic regression (univariate analysis), the results showed that for both abdominal colic and constipation, female gender was observed significantly with low prevalence than male with the odd ratios and *p*-value of (OR 0.54, *p* 0.01) and (OR 0.57, *p* 0.05) respectively. However, for influenza, the self-medication prevalence (OR 1.86, *p* = 0.006) observed was significantly higher in female participants (Table 3).

**Table 2:** Self-medication prevalence among university students (*N* = 373).

**Conditions for self-medication**	**Prevalence** ***n* (%)**	**Male (*n* = 152)** ***n* (%)**	**Female (*n* = 221)** ***n* (%)**	***p*-value**
Headache	346 (92.8)	145 (95.3)	201 (90.9)	0.104
Pain	107 (28.7)	51 (33.5)	56 (25.3)	0.085
Fever	111 (29.8)	42 (27.6)	69 (31.2)	0.456
Colic	119 (31.9)	37 (24.3)	82 (37.1)	0.009^*^
Cough	140 (37.5)	57 (37.5)	83 (37.5)	0.991
Hair health	96 (25.7)	37 (24.3)	59 (26.7)	0.609
Skin health	107 (28.7)	40 (26.3)	67 (30.3)	0.401
Drowsiness	60 (16.1)	26 (17.1)	34 (15.3)	0.657
Influenza	113 (30.3)	58 (38.1)	55 (24.9)	0.006^*^
Vomiting	47 (12.6)	16 (10.5)	31 (14.02)	0.317
Diarrhea	74 (19.8)	28 (18.4)	46 (20.8)	0.569
Constipation	66 (17.7)	20 (13.1)	46 (20.8)	0.057^*^

* Chi-square test, p-value * ≤ 0.05.

**Table 3:** Gender related prevalence of self-medication conditions among the study population (*n* = 373).

**Variable**	**Conditions prevalence for self-medication (*n*%)**	**Univariate analysis**	
		**OR (95% CI)**	***p*-value**
^*^Gender	**Headache (*****n*** **= 346, 92.8%)**		
Male	145 (95.3)	1	
Female	201 (90.9)	2.06 (0.84–5.003)	0.11
	**Pain (*****n*** **= 107, 28.7%)**		
Male	51 (33.5)	1	
Female	56 (25.3)	1.48 (0.94–2.34)	0.08
	**Fever (*****n*** **= 111, 29.8%)**		
Male	42 (27.6)	1	
Female	69 (31.2)	0.84 (0.53–1.32)	0.84
	**Colic (*****n*** **= 119, 31.9%)**		
Male	37 (24.3)	1	
Female	82 (37.1)	0.54 (0.34–0.86)	0.01^*^
	**Cough (*****n*** **= 140, 37.5%)**		
Male	57 (37.5)	1	
Female	83 (37.5)	0.99 (0.65–1.52)	0.99
	**Hair health (*****n*** **= 96, 25.7%)**		
Male	37 (24.3)	1	
Female	59 (26.7)	0.88 (0.54–1.42)	0.60
	**Skin health (*****n*** **= 107, 28.7%)**		
Male	40 (26.3)	1	
Female	67 (30.3)	0.82 (0.51–1.30)	0.40
	**Drowsiness (*****n*** **= 60, 16.1%)**		
Male	26 (17.1)	1	
Female	34 (15.3)	1.13 (0.64 – 1.98)	0.65
	**Influenza (*****n*** **= 113, 30.3%)**		
Male	58 (38.1)	1	
Female	55 (24.9)	1.86 (1.19–2.91)	0.006^*^
	**Vomiting (*****n*** **= 47, 12.6%)**		
Male	16 (10.5)	1	
Female	31 (14.02)	0.72 (0.37–1.37)	0.31
	**Diarrhea (*****n*** **= 74, 19.8%)**		
Male	28 (18.4)	1	
Female	46 (20.8)	0.85 (0.50–1.44)	0.56
	**Constipation (*****n*** **= 66, 17.7%)**		
Male	20 (13.1)	1	
Female	46 (20.8)	0.57 (0.32–1.02)	0.05^*^

* Binary logistic regression (univariate analysis), p-value * ≤ 0.05, Male (n = 152), Female (n = 221).

### Influential Factors in Self-Medication

The most common justifications for university students to indulge in self-medication were “mild illness” and “time-saving option” where followed by other options that was urgency to self-medicate and cost-effectiveness. There was a statistically significant difference between the genders regarding the reasons for self-medication (*p* = 0.011; Table 4). Women identified more influential factors than did men.

**Table 4:** Factors in self-medication (*N* = 373).

**Factors in self-medication**	**Male (*n* = 152)** ***n* (%)**	**Female (*n* = 221)** ***n* (%)**	***p*-value**
Mild illness	108 (71.05)	121 (54.75)	0.011^*^
Time-saving option	24 (15.8)	58 (26.24)	
Urgency	15 (9.8)	36 (16.2)	
Cost-effectiveness	5 (3.2)	6 (2.7)	

* Chi-square test, p-value * ≤ 0.05.

### Pharmacists' Role in Self-Medication

Table 5 presents the data on pharmacist consultations for self-medication and as a drug consultant among University of Hail students. The results of the chi-square test indicated that the university program had a significant influence on the participants who sought pharmacists' opinions when taking more than one drug (76.4%, *n* =285; *p* = 0.010; Table 5). A statistically significant difference was observed between the academic program and pharmacist consultations regarding increasing the duration of drug consumption (*n* = 244, 65.4%; *p* = 0.011). The chi-square test indicated a lack of statistical significance between age and pharmacist consultation.

**Table 5:** Pharmacists' role in self-medication among students (*N* = 373).

**Pharmacist consultation**	**University Program** ***n*** **(%)**						***p*-value**
	**Foundation course [*****n*** **=8 4 (22.5)]**		**Undergraduate [*****n*** **= 282 (75.6)]**		**Post-graduate [*****n*** **= 7 (1.8)]**		
	**Yes**	**No**	**Yes**	**No**	**Yes**	**No**	
Consult a pharmacist before taking a drug	63 75%	21 25%	201 71.3%	81 28.7%	4 57.1%	3 42.8%	0.547
Ask a pharmacist about the recommended dosage	66 78.5%	18 21.4%	226 80.1%	56 19.9%	5 71.4%	2 28.5%	0.821
Consult pharmacist about taking the drug for a longer time	59 70.2%	25 29.8%	184 65.2%	98 34.7%	1 14.3%	6 85.7%	0.011^*^
Report occurrence of side-effects to pharmacist	61 72.6%	23 27.4%	193 68.4%	89 31.6%	2 28.6%	5 71.4%	0.054^*^
Inform the pharmacist about drugs being taken	61 72.6%	23 27.4%	195 69.1%	87 30.8%	3 42.8%	4 57.1%	0.254
Seek a pharmacist's opinion when taking more than one drug	64 76.2%	20 23.8%	219 77.6%	63 22.3%	2 28.6%	5 71.4%	0.010^*^

* Chi-square test, p-value * ≤ 0.05.

## Discussion

The appropriate use of self-medication, with full knowledge about the drugs and the condition to be treated, could be beneficial for mild illnesses. However, the risks cannot be ignored. They include dosage errors and the inappropriate duration of drug use. In addition, the occurrence of drug–drug interactions could negatively affect health.

This study was conducted in the Hail region to estimate the incidence and prevalence of self-medication among University of Hail students. The incidence of self-medication among the students was very high. In a study in Al-Qassim Province in Saudi Arabia, Saeed et al. reported that 86.2% of university students were self-medicated. However, at Taibah University Medina, 64.8% of the students were practiced self-medication (10, 11). According to Albusalih et al., the self-medication prevalence in the medicine and pharmacy colleges in Dammam city was 26%, the lowest in Saudi medical colleges (16). The high prevalence of self-medication is not unique to Saudi Arabia. A study conducted in Rio Grande, Brazil indicated that 86.4% of the students were self-medicated. Zafar et al. found that 76% of university students in Karachi, Pakistan were self-medicated (14, 17). The prevalence among Palestinian university students was 98%, which is the highest (9). In Slovenia, 92.3% of Ljubljana University students were found to be self-medicated (7). In Oman, 94% of university students were self-medicated. However, in Islamabad, Pakistan, the prevalence of self-medication among university students was 42% (18, 19).

In the current study, all 373 participants reported being self-medicated. The prevalence was higher among male students where 148 (97.3%) male student self-medicate out total 152 students participated in the study. Out of total 221 female students participated in the study survey, 207 (93.6%) used self-medication. In a study of students at Taibah University in Madinah, Saudi Arabia, Aljaouni et al. found that 65.5% of self-medicated students were women. In Oman, Egypt and Ethiopia, the prevalence was higher among women than men; thus, gender could be a factor in self-medication (11, 18, 20, 21).

The results of the present study (Table 2) support those of Saeed et al. in Dammam and Zafar et al. in Karachi regarding headaches being the most reported reason (59.9 and 72.4%, respectively) for seeking self-medication (14, 16). According to Alshogran et al., the most common indication (81.9%) for self-medication among students in Jordan was headaches (22). Because of the availability of free healthcare services in Saudi Arabia, the high prevalence of self-medication could be patients' believing that these diseases are mild and do not require treatment by a physician. Pharmacists are not only drug suppliers and distributors; they are also healthcare providers. They can play a major role in patients' decisions about self-medication by providing information. Thus, the focus should be on disease management and survival rather than sales and profit.

Especially for over-the-counter products, pharmacists can play a very important role by providing patients with the necessary information about medications and dosage. Their role in patient care services should include providing advice, building awareness about medical products and providing detailed information about medication effects, dosage and duration and possible side effects and drug–drug interactions, in addition to monitoring patients. The present study found that 65% to 79% of the university students practicing self-medication sought the assistance of a pharmacist. This suggests pharmacists' high confidence in the community and awareness of their active role in health care.

The results indicated that the students' main reasons for self-medication were as follows: mild illness (61%), time-saving option (22%), urgency (13%) and cost-effectiveness (2.9%). A study at a Dammam public university found that 35.1% of the medical and pharmacy students sought self-medication for mild problems (16). However, Sharif et al. reported that 72% of Sharjah university students self-medicated for mild disease (23).

It was assumed that the prevalence of self-medication among medical college students would be lower than that among other students because of their awareness of the drugs and their side effects. However, the results indicated that there was no difference between the medical and non-medical students. In a Jordanian university, the prevalence among medical and non-medical students was similar (22). The percentage of students who consulted pharmacists about drugs for self-medication (Table 3) revealed the students' awareness of the pharmacist's important role and their concerns about the drugs they had sought or used. The results indicated that 79.6% of the students sought pharmacist recommendations; thus, they were aware of dose variations. More women (59.2%) than men (40.8%) consulted pharmacists before taking drugs. Most (76.4%) of the students sought a pharmacist's advice about taking more than one drug; thus, they were aware of drug interactions.

## Conclusion

The prevalence of self-medication among the university students and the pharmacists' contribution to this behavior were high. The occurrence of self-medication is concerning. Pharmacists can play a significant role by providing advice on rational options, increasing awareness about the care to be taken in medicine use, providing data on side effects and examining patients for their illnesses. Healthcare organizers should educate the public about the negative effects of irresponsible self-medication.

### Limitation

The questionnaire was self-reported and this could have led to under or over reporting of the self-medication practices. Furthermore, the study sample size calculation was through a convenient sampling technique, which is inferior to probability sampling in its representativeness. Moreover, the sample size of the study was not so large to generalize the results over the whole kingdom population.

### Recommendation

Implications of the awareness and education regarding the self-medication practices can be useful in future.

## Data Availability Statement

The original contributions presented in the study are included in the article/Supplementary Material, further inquiries can be directed to the corresponding author.

## Author Contributions

FA contributed to the study conceptualization, data curation, methodology and project administration, and manuscript editing. AAlo contributed to formal analysis, manuscript revision, and resources. AAls contributed to formal analysis, investigation, and data curation. AAlh contrinuted to formal analysis, data collection, investigation, and software. AAlra contributed to the data collection, formal analysis, writing, and revision. AAlm contributed to the methodology, study design, and manuscript revision. AAlre contributed to the data collection, software, data retrieving, and writing. KK is the scientific coordinator of this study and has developed the concept and made substantive intellectual contributions to the manuscript. All authors contributed to the study concept and design, manuscript revision for intellectual concepts and final approval for manuscript submission.

## Conflict of Interest

The authors declare that the research was conducted in the absence of any commercial or financial relationships that could be construed as a potential conflict of interest.

## Publisher's Note

All claims expressed in this article are solely those of the authors and do not necessarily represent those of their affiliated organizations, or those of the publisher, the editors and the reviewers. Any product that may be evaluated in this article, or claim that may be made by its manufacturer, is not guaranteed or endorsed by the publisher.
