# Supplementary material for: University Students' Self-Medication Practices and Pharmacists' Role: A Cross-Sectional Survey in Hail, Saudi Arabia
Source: Front Public Health. 2021 Dec 17;9:779107. doi: 10.3389/fpubh.2021.779107 (PMC8718403; doi:10.3389/fpubh.2021.779107)
Supplement: Supplementary file 1 [file Data_Sheet_1.docx]

**Appendix 1**

**DATA COLLECTION FORM & SURVEY QUESTIONNAIRE**

University students’ self-medication practices and pharmacists’ roles: A cross-sectional survey in Hail, Saudi Arabia

**SECTION 1. RESPONDENT DEMOGRAPHICS**

| **S.NO** | **RESPONDENTS DEMOGRAPHICS** | **RESPONSE** | | | | | | | | | |
| --- | --- | --- | --- | --- | --- | --- | --- | --- | --- | --- | --- |
| **1** | **ID NUMBER** |  | | | | | | | | | |
| **2** | **AGE GROUP** | <20 | 21-25 | | | | 26-30 | | | | >30 |
| **3** | **GENDER** | MALE | | | | | FEMALE | | | | |
| **4** | **SMOKING** | YES | | | | | NO | | | | |
|  | **NATIONALITY** | SAUDI | NON- SAUDI | | | | OTHER | | | | |
| **5** | **DRUG ALLERGY** | YES | | | | | NO | | | | |
| **6** | **UNIVERSITY PROGRAMME** | FOUNDATION | | UNDER GRAD | | | | | | POST GRAD | |
|  | **UNIVERSITY COURSE** | ARTS AND SOCIAL SCIENCE | HOSPITAL FOOD AND LEISURE MANAGEMENT | | | HEALTH AND MEDICAL SCIENCE | | | BUSINESS AND LAW | | |
|  |  | ENGENERING | ARCHITECTURE | | | | | TECHNOLOGY AND DESIGN | | | |
| **7** | **RESIDENCE** | DAY SCHOLAR | | | HOSTELER | | | | | | |
| **8** | **DO YOU SELF MEDICATE** | YES | | | NO | | | | | | |

**SECTION 2.**

**2a. CONDITIONS FOR SELF-MEDICATION IS PRACTICED**

| **VARIABLES** | **RESPONSE** | **RESPONSE** |
| --- | --- | --- |
| **Headache** | Yes | No |
| **Pain** | Yes | No |
| **Fever** | Yes | No |
| **Abdominal colic** | Yes | No |
| **Cough** | Yes | No |
| **Hair health** | Yes | No |
| **Skin health** | Yes | No |
| **Drowsiness** | Yes | No |
| **Flue** | Yes | No |
| **Vomiting** | Yes | No |
| **Diarrhea** | Yes | No |
| **Constipation** | Yes | No |

**2b. FACTORS FOR SELF-MEDICATION**

| **STATEMENTS** | **RESPONSE** | **RESPONE** |
| --- | --- | --- |
| **Mild nature of illness** | Yes | No |
| **Cost effectiveness** | Yes | No |
| **Time saving** | Yes | No |
| **Urgency** | Yes | No |
| **Other (Please specify)** |  |  |

**SECTION 3. PHARMACIST CONTRIBUTION TOWARDS SELF-MEDICATION**

| **STATEMENTS** | **RESPONSE** | **RESPONE** |
| --- | --- | --- |
| **Do you consult pharmacist before taking a drug** | Yes | No |
| **Ask pharmacist about drug recommended dose** | Yes | No |
| **Consult pharmacist for taking the drug for a longer period of time** | Yes | No |
| **Report incidence of drug side effects to pharmacist** | Yes | No |
| **Inform the pharmacist about drugs being taken** | Yes | No |
| **Request pharmacist opinion when taking one or more drugs simultaneously** | Yes | No |
